# Integrated transcriptomic analysis reveals transcriptional changes associated with begomovirus infection in the medicinal plant *Emilia sonchifolia*

**DOI:** 10.3389/fpls.2026.1827690

**Published:** 2026-05-26

**Authors:** Duoduo Wang, Hui Ling, Yupeng Chen, Tingting Liu, Jiyuan Wang, Yiyong Zheng, Wenting Chen, Xujia Ning, Zhenling Wu, Daogang Guan, Yuxi Liu, Xinchao Xu

**Affiliations:** 1 Department of Agriculture, Nutrition, and Food Systems, University of New Hampshire, Durham, NH, United States; 2 Guangxi Key Laboratory of Smart Agriculture for Special Horticultural Crops/Guangxi Zhuang Autonomous Region Engineering Research Center of Facility Agriculture, College of Smart Agriculture, Yulin Normal University, Yulin, China; 3 Department of Biochemistry and Molecular Biology, School of Basic Medical Sciences, Southern Medical University, Guangzhou, China; 4 Boluo Institute of Agricultural Science, Huizhou, China; 5 Alfred E. Mann Department of Biomedical Engineering, University of Southern California, Los Angeles, CA, United States; 6 Department of Traditional Chinese Medicine and Gynecology, Shunde Women and Children’s Hospital of Guangdong Medical University, Foshan, China

**Keywords:** begomovirus, *Emilia sonchifolia*, Emilia yellow vein virus Gx (EYVV-Gx), transcriptome, integrated transcriptomic analysis, plant defense, RNA-seq

## Abstract

**Introduction:**

*Emilia sonchifolia* L. (*E. sonchifolia*) is a traditionally medicinal plant widespread in tropical and subtropical regions and is valued for its diverse bioactive properties, including anti-inflammatory, antioxidant, and anti-tumor activities. In October 2021, two groups of *E. sonchifolia*—one non-symptomatic (Mock) and the other exhibiting yellow vein symptoms (Vln)—were identified from an experimental nursery in Yulin City, Guangxi Zhuang Autonomous Region, China. The virus infecting symptomatic plants was isolated, sequenced, and confirmed to be closely related to Emilia yellow vein virus Fz1 (EYVV-Fz1) from Fujian province, and was designated Emilia yellow vein virus Gx (EYVV-Gx).

**Methods:**

To reveal gene expression changes and enriched biological pathways in response to begomovirus infection in the medicinal plant *E. sonchifolia*, transcriptome analysis between Mock and Vln plants was conducted using integrated Single Molecule Real Time (SMRT)-seq and Illumina-seq technologies.

**Results:**

A total of 195 differentially expressed genes (DEGs) were detected, and enriched in pathways related to reactive oxygen species (ROS) scavenging, cell wall remodeling, hormone signaling, transcriptional regulation, as well as stress and immune related responses. Reverse transcription quantitative polymerase chain reaction (RT-qPCR) validations revealed that gene expression was group-dependent and tissue-specific.

**Discussion:**

These findings provide new insights into infection-associated transcriptional responses of *E. sonchifolia* under natural begomovirus infection and represent the first transcriptomic analysis of this species based on the combined use of Illumina and SMRT sequencing. This study provides a valuable resource for future molecular studies and may support improvements in cultivation strategies for medicinal plants.

## 1 Introduction

*Emilia sonchifolia* (L.) (*E. sonchifolia*) is an annual herbaceous plant in the *Asteraceae* family and is widely distributed across tropical and subtropical regions (Huang et al., 2025). It is also a traditional medicinal plant whose extracts, derived from aerial parts (primarily leaves and stems), exhibit diverse bioactive properties, including anti-inflammatory, anti-tumor, and antioxidant effects (Gilcy and Kuttan, 2015; Sophia et al., 2011; George and Kuttan, 2016). *E. sonchifolia* also plays a significant ecological role. It is often found in disturbed environments such as roadsides, fields, and waste areas, where it can thrive and remain resilient to stresses. The plant contributes to soil stabilization and erosion control, and provides shelter for pollinators. This ecological adaptability makes *E. sonchifolia* a valuable component in agroecosystems and natural landscapes.

However, its growth and development are frequently threatened by biotic stresses, particularly geminiviruses, in several countries, including Thailand (Zhao et al., 2018). Several geminivirus strains have been identified in China, such as Ageratum yellow vein China virus (AYVCNV; Guangxi, KJ016239), Tobacco curly shoot virus (TCSV; Yunnan, MN481164), and multiple isolates of Emilia yellow vein virus (EYVV) from Fujian (EU377539, JQ247188), Guangxi (KJ016240), and Yunnan (MN201995, MN201996, MN2019957, MN201998, MN2019959) (Li et al., 2022).

According to the International Committee on Taxonomy of Viruses (ICTV), *begomovirus* is the largest genus within the *Geminiviridae* family, which encompasses fourteen genera (Fiallo-Olivé et al., 2021). Begomoviruses are characterized by their geminate isometric virions containing small, circular, single-stranded DNA (ssDNA) genomes encapsulated within twin virions (Zhou, 2013). They can be further classified as monopartite or bipartite viruses. Monopartite viruses contain a single ssDNA genome, whereas bipartite viruses harbor two genomic components, designated DNA-A and DNA-B (Briddon et al., 2010). Monopartite begomoviruses are often associated with betasatellites or alphasatellites, each approximately half the size of the begomoviruse genome (Zhou, 2013). Begomovirus evolution is primarily driven by gene recombination among strains, which can enhance virulence, broaden host range, and facilitate environmental adaptation (Fiallo-Olivé and Navas-Castillo, 2023; Qadir et al., 2019; Xie et al., 2013). Begomoviruses are primarily transmitted by whiteflies and can infect a wide range of plants, causing significant economic losses in many crops worldwide, with yield reductions reported to reach up to 100% under severe infection conditions (Cana-Quijada et al., 2020).

Transcriptome analysis is a powerful approach for elucidating molecular mechanisms underlying plant antiviral defenses. Next-generation sequencing (NGS) technologies, particularly Illumina platforms, generate short reads and have been widely used to analyze genome-wide gene expression changes. However, NGS can generate gaps due to short read lengths. The recent development of single-molecule real-time (SMRT) sequencing by the PacBio platform enables the sequencing of long transcripts up to 20 kb, overcoming the gaps generated by short-read sequencing (van Dijk et al., 2023). SMRT sequencing also facilitates the identification of novel isoforms, long non-coding RNAs (lncRNAs), alternative splicing events, gene fusions, and DNA methylation. However, SMRT sequencing exhibits a higher error rate (~10%) due to lower sequencing depth compared to Illumina RNA-seq (Khiste and Ilie, 2017). Integrating SMRT and Illumina sequencing can overcome the limitations of each technology, producing higher-quality *de novo* transcriptome assemblies. This integrative approach has been used to reveal molecular mechanisms of plant defense against biotic stresses (Zhu et al., 2018; Ji et al., 2025; Mei et al., 2020).

Plant responses to virus infection involve a complex interplay of various mechanisms and pathways, including hormonal signaling, reactive oxygen species (ROS) regulation, epigenetic regulation, and activation of resistance-related genes (Jiang et al., 2025). These processes collectively contribute to the modulation of host responses to infection. Understanding antiviral mechanisms in medicinal plants is crucial for discovering bioactive compounds with potential therapeutic properties, as well as for developing novel strategies to enhance crop resistance to viral diseases (Adeosun and Loots, 2024). While much of the research in this area has focused on model plants, there is a growing recognition of the need to explore antiviral responses in non-model species, particularly those with important medicinal or agricultural value.

To date, the molecular mechanisms underlying the responses of *E. sonchifolia* to geminivirus infection remain unclear. In 2021, *E. sonchifolia* plants exhibiting typical geminivirus symptoms, along with asymptomatic plants, were identified in an experimental nursery in Yulin City, Guangxi Zhuang Autonomous Region, China. The causal agent was identified as EYVV-Gx. Symptomatic plants were designated as Vln, while symptomless plants from the same field population were designated as Mock controls. Comparative transcriptome analysis between Vln and Mock plants was performed using integrated Illumina and SMRT sequencing to investigate infection-associated transcriptional responses. Because these plants are not genetically defined as resistant or susceptible lines, the observed differences were interpreted as infection-associated responses rather than resistance mechanisms. This study provides the first genome-wide transcriptomic comparison of *E. sonchifolia* under EYVV infection. The results indicate that pathways related to DNA replication, ROS regulation, and cell wall remodeling are associated with host responses to infection. These findings provide a transcriptomic resource for future functional studies and may contribute to improving the understanding of plant–virus interactions in related species.

## 2 Materials and methods

### 2.1 Plant material

*E. sonchifolia* plants were collected in October 2021 from an experimental nursery located on the campus of Yulin Normal University in the Yulin, Guangxi Autonomous Region, China. The plants were locally occurring, field-grown individuals maintained in the experimental nursery. The species is not listed as protected or endangered, and no specific permissions or licenses were required for the collection of this plant material. All field sampling and experimental research on these plants were conducted in accordance with local regulations, as well as institutional, national, and international guidelines for plant research. Plant species identification was performed based on standard morphological characteristics. No voucher specimen was deposited, as the samples were collected exclusively for molecular analyses. A total of ten plants were collected for this study, including five virus-infected plants (designated as Vln) displaying typical yellow vein symptoms, and five asymptomatic plants (designated as Mock), which showed no visible symptoms of yellow vein disease. Vln plants were collected from the field at the two-true-leaf stage and were displaying typical yellow vein symptoms. Asymptomatic plants were collected from the same experimental nursery at the same developmental stage. Both Vln and Mock plants were transferred to individual greenhouse compartments with controlled temperature and humidity to mimic further exposure to field-borne viruses. These controlled conditions were identical for both groups, ensuring that any differences in their responses were due to viral infection rather than environmental factors. During this period, Vln plants continued to display typical viral symptoms, whereas Mock plants remained asymptomatic. Tissue samples (leaves, stems, and flowers) were collected from both groups at comparable developmental stages. Leaf tissues at the 4–5 leaf stage were used for transcriptome analysis, while stem and flower tissues were collected accordingly. All samples were immediately frozen in liquid nitrogen and stored at −80 °C until further analysis. Asymptomatic Mock plants served as controls for comparison with Vln plants.

### 2.2 RNA extraction, cDNA library construction, and sequencing using PacBio sequel and Illumina platforms

Total RNA was extracted from leaf tissues using the PureLink™ Plant RNA Reagent (Invitrogen, Carlsbad, CA, USA). RNA degradation and contamination were assessed using 1% agarose gel electrophoresis. RNA concentration and purity were measured using a NanoDrop™ 2000 spectrophotometer. RNA samples used for library construction met the following criteria: total RNA≥1ug, concentration≥200ng/μL, and an OD260/OD280 ratio between 1.8 and 2.2. RNA integrity was further evaluated using an Agilent 2100 Bioanalyzer with the following thresholds: 28S:18S ratio ≥ 1.4 and RNA integrity number (RIN) ≥ 8. For PacBio Iso-Seq analysis, total RNA from ten independent biological samples (five Vln and five Mock plants) was pooled in equal amounts. One microgram of pooled RNA was used for full-length cDNA synthesis using the Clontech SMARTer™ PCR cDNA Synthesis Kit (Clontech, Mountain View, CA, USA). mRNA was reverse transcribed into first-strand cDNA using poly-T primers, and second-strand cDNA was synthesized. Size selection was carried out using a BluePippin (Sage Science, Beverly, MA, USA) and the 1–2 kb, 2–3 kb, 3–6 kb, and 5–10 kb cDNA fractions were collected. After size selection, the collected cDNA fractions were treated with a DNA damage repair mix, followed by end repair. A PacBio library was constructed using the PacBio SMRTbell Template Prep Kit (Pacific Biosciences, Menlo Park, CA, USA). SMRT sequencing was then performed on the PacBio RS II system (Pacific Biosciences). For Illumina RNA-seq analysis, RNA-seq libraries were constructed independently for each of the ten samples (five Vln and five Mock plants) using the NEB Next Ultra RNA Library Prep Kit for Illumina following the manufacturer’s instructions. Libraries were sequenced in 150 bp paired-end mode using an Illumina HiSeq X Ten.

### 2.3 High-quality transcriptome assembly using SMRT sequencing and error correction with Illumina reads

Polymerase reads were processed into subreads by removing adapter sequences and low-quality reads using SMRT Link 9.0.0. Circular consensus sequences (CCS) were generated using the ToFU pipeline with default parameters. Full-length and non-full-length cDNA reads, including both full-length non-chimeric and chimeric reads, were classified based on the presence and position of the 3’ primer, 5’ primer, and poly(A) tail. Full-length non-concatemer (FLNC) sequences were identified by filtering out non-full-length cDNA reads from the CCS reads. The Iterative Clustering for Error Correction (ICE) algorithm was then applied to cluster the FLNC sequences based on sequence similarity, producing a consensus transcript (unique isoform) for each cluster. The resulting full-length consensus sequences from ICE were polished using Quiver (Chin et al., 2013) and further error-corrected with Illumina short reads using the LoRDEC method (Salmela and Rivals, 2014), resulting in transcripts with a predicted accuracy exceeding 99%. Afterward, CD-HIT 4.8.1 (Fu et al., 2012) was used to remove redundant high-quality full-length transcripts. The resulting non-redundant, high-quality transcripts were used as the reference transcriptome for downstream analyses.

### 2.4 Coding sequence prediction and functional annotation

The protein-coding regions within the transcript sequences were predicted using TransDecoder (version 3.0.0) with default parameters (Haas et al., 2013). Functional annotation of all predicted coding DNA sequences (CDSs) were subsequently conducted using Diamond BLASTx v2.0.8.146 (Buchfink et al., 2015) by aligning against multiple databases, including the National Center for Biotechnology Information non-redundant (NCBI nr) database, Swiss-Prot, Translated European Molecular Biology Laboratory (TrEMBL), Gene Ontology (GO), Kyoto Encyclopedia of Genes and Genomes (KEGG), Protein families (Pfam), and Evolutionary Genealogy of Genes: Non-supervised Orthologous Groups (eggNOG).

### 2.5 Prediction of long non-coding RNAs, alternative splicing and simple sequence repeats

Long non-coding RNAs (lncRNAs) from isoforms that were not annotated by any of the five protein databases were predicted using four tools, including Coding-Non-Coding Index (CNCI2) (Luo et al., 2017), Coding Potential Calculator 2 (v1.0.1) (Kong et al., 2007), Predictor of lncRNAs and mRNAs based on an improved k-mer scheme (PLEK 1.2) (Li et al., 2014), and Coding Potential Assessment Tool (CPAT 1.2.4) (Wang et al., 2013). Simple sequence repeats (SSRs) in the transcriptome were identified using Microsatellite (MISA) tool with default parameters as follows: sequences defined as microsatellites must contain at least ten mononucleotide repeats, six dinucleotide repeats, five trinucleotide repeats, five tetranucleotide repeats, five pentanucleotide repeats, and five hexanucleotide repeats (Beier et al., 2017). Alternative splicing events were identified based on combined short-read RNA-seq and SMRT sequencing data using IsoSeq_AS_de_novo v1.0 (Liu et al., 2017).

### 2.6 Advanced protein prediction and functional analysis

The transmembrane Hidden Markov Model (TMHMM) Server v2.0 was used to predict the transmembrane helices (Krogh et al., 2001). The SignalP 4.1 Server was used to predict the signal peptide sequences (Petersen et al., 2011). Subcellular localizations of the proteins were predicted by DeepLoc-2.0, a multi-label predictor that can differentiate between 10 localizations: nucleus, cytoplasm, extracellular, mitochondrion, cell membrane, endoplasmic reticulum, chloroplast, Golgi apparatus, lysosome/vacuole, and peroxisome (Thumuluri et al., 2022). Transcription factors (TFs) were predicted by performing BLASTP searches against the Plant Transcription Factor Database. Resistance (R) genes were predicted by performing BLASTP searches against PRGdb 4.0, an updated database dedicated to genes involved in the plant disease resistance process (Calle García et al., 2022).

### 2.7 Identification and functional characterization of differentially expressed genes

The Illumina cleaned reads were mapped to the assembled reference transcriptome using Bowtie2 (Langmead and Salzberg, 2012). Gene expression levels were estimated using RSEM (Li and Dewey, 2011), which generated both raw read counts and normalized expression values (FPKM). Raw read counts were used as input for differential expression analysis using DESeq2 (version 1.18.1), which performs internal normalization based on size factors (Anders and Huber, 2010). FPKM values were used for expression quantification and visualization. *P*-values from multiple hypothesis testing were adjusted using the Benjamini and Hochberg’s method to control the false discovery rate (FDR). Differentially expressed genes (DEGs) with an FDR < 0.05 and |log_2_ fold change| > 1 were considered significant. Hierarchical clustering of DEGs for heatmap visualization was performed using Euclidean distance and complete linkage, and expression values were normalized as Z-scores. GO and KEGG pathway enrichment analyses were performed using the clusterProfiler R package. Pathways were considered significantly enriched when the adjusted *p*-value (padj) was < 0.05.

### 2.8 Validation of DEGs using reverse transcription quantitative polymerase chain reaction

Nine DEGs were selected to compare their expression levels in leaf tissues between Mock and Vln plants, as well as among different tissues in Vln plants, including flowers, stems, and leaves. Total RNA was isolated using TRIzol reagent. RNA quality was checked via agarose gel electrophoresis, and the concentration and purity were examined using a Nano-400A ultramicrospectrophotometer. For each sample, 500 ng of total RNA was used for first-strand cDNA synthesis with the HiScript II Q RT SuperMix kit (#R223-01, Vazyme). Reverse transcription quantitative polymerase chain reaction (RT-qPCR) reactions were prepared using ChamQ SYBR qPCR Master Mix (Vazyme, Nanjing, China) and performed on an Applied Biosystems™ QuantStudio™ 6 Flex Real-Time PCR System (Applied Biosystems, Foster City, CA, USA) using 96-well plates. The cycling conditions were as follows: 95 °C for 30 s, followed by 40 cycles of 95 °C for 10 s and 60 °C for 30 s. A melting curve analysis was performed from 65 °C to 95 °C to confirm amplification specificity. The *glyceraldehyde 3-phosphate dehydrogenase* (*GAPDH*) gene was used as the reference control. Relative gene expression was calculated using the 2^−ΔΔCT^ method. All primers used in this study are listed in **Supplementary Table 1**. Five biological replicates per group (three technical replicates each) were used for comparison between Mock and Vln leaf tissues, and statistical significance was assessed using Student’s t-test (*p* < 0.05). For tissue-specific expression analysis in Vln plants, three biological replicates per tissue (three technical replicates each) were used, and significance was assessed using one-way ANOVA followed by Tukey’s *post-hoc* test (*p* < 0.05).

### 2.9 Characterization and validation of EYVV in *E. Sonchifolia* using small RNA sequencing and PCR cloning

The viral strain infecting *E. sonchifolia* was analyzed using small RNA sequencing of infected leaf tissues from Vln plants, coupled with Sanger sequencing of PCR-amplified viral genome. RNA from five individual Vln plants was isolated using TRIzol^®^ Reagent (Invitrogen). A total of 1 μg RNA, pooled from five biological replicates of Vln plants, was used to construct a single small RNA library using the TruSeq Small RNA Library Preparation Kit (Illumina, CA, USA). RNA was quantified and size-fractionated via 15% polyacrylamide gel electrophoresis (PAGE) to enrich small RNAs in the range of 18 to 32 nt. The purified small RNAs were ligated with 5′and 3′adapters, and these ligated RNAs were used as templates for cDNA synthesis. After amplification and purification, the library was deep-sequenced on the HiSeq 2000 SE50 platform (Majorbio, Shanghai, China).

Quality control of raw data was performed using fastp, followed by assembly using velvet_1.2.10. The assembled contigs were then subjected to a BLAST search against the Descriptions of Plant Viruses (DPV) database (http://www.dpvweb.net/) (Adams and Antoniw, 2006). Virus-induced small interfering RNA (siRNA) was predicted and analyzed for the identified virus. To validate the viral genome, total DNA was extracted from both Vln plants and Mock plants using the CTAB method. The viral genome was PCR-amplified with Vazyme LAmp DNA Polymerase (P301, Vazyme, Nanjing, China), cloned into the T-Vector pMD™19 (3271, Takara, Beijing, China), and confirmed by Sanger sequencing. Primers used for amplifying the viral genome were designed based on the virus reference genome obtained from NCBI and are listed in **Supplementary Table 1**.

## 3 Results

### 3.1 Identification and characterization of EYVV in *E. Sonchifolia*

**Figure 1:**
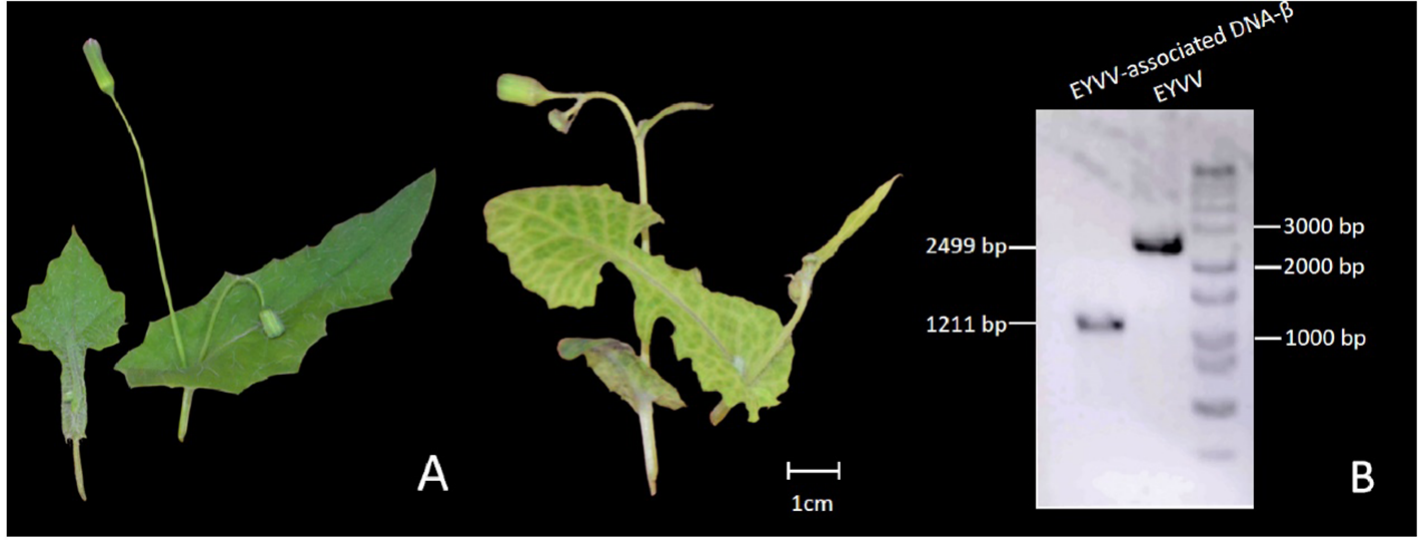
Phenotypic symptoms and viral genome confirmation in *Emilia sonchifolia E.* (*sonchifolia*). **(A)** Comparison of Mock (left) and Vln (right) *E. sonchifolia* plants. Mock plants show no visible symptoms, whereas Vln plants exhibit characteristic yellow vein symptoms. Scale bar = 1 cm. Experimental conditions: Mock and Vln plants were grown under identical environmental conditions described in the Materials and Methods section. **(B)** PCR amplification of viral genome. DNA was extracted from leaves of Mock- and Vln plants. The lane labeled “EYVV-associated DNA-β” shows the betasatellite DNA component (~1,211 bp); the lane labeled “EYWV” shows the viral genome (~2,499 bp); the rightmost lane contains a DNA ladder.; The presence of virus-specific bands confirms viral infection in Vln plants.

Representative leaf phenotypes of Mock and Vln plants are shown in **Figure 1A**. To characterize the virus, small RNAs were isolated from Vln leaves and sequenced using an Illumina deep-sequencing platform. A total of 10,198,692 raw reads were obtained. Low-quality reads, including sequences shorter than 18 nt or longer than 32 nt, were removed, resulting in 7,941,706 clean reads. Analysis of read-length distribution showed that 24-nt sRNAs were the most abundant (26.95%), followed by 22-nt (15.6%) and 21-nt (15.06%) sRNAs. The clean reads were assembled using velvet (1.2.10), generating 4,055 contigs ranging from 32 nt to 926 nt in length. Because small RNA reads are short (typically 21–24 nt), BLAST analysis may detect sequence similarity to several related viruses; such matches reflect conserved regions among begomoviruses and do not necessarily indicate co-infection. BLASTn analysis of the assembled contigs against the DPV database revealed the highest sequence identity with EYVV-Fz1, a member of the genus Begomovirus in the family Geminiviridae. The presence of EYVV was further confirmed by PCR amplification and Sanger sequencing of the viral genome. The complete viral genome was PCR-amplified using primers designed based on the EYVV-Fz1 reference genome retrieved from NCBI and sequenced by Sanger sequencing. PCR amplification produced the expected viral fragment in Vln plants, whereas no amplification was detected in Mock plants, confirming the absence of EYVV infection in the Mock samples. Sequence analysis of Vln plants revealed a 2.7-kb DNA fragment corresponding to the monopartite begomovirus genome and an additional fragment approximately half this size corresponding to the associated betasatellite (**Figure 1B**). The original gel image showing the absence of amplification in Mock samples is provided in **Supplementary Figure 1**. The complete viral genome infecting *E. sonchifolia* was sequenced and subjected to BLASTn analysis against the NCBI nucleotide database. The viral genome (2,725 bp) showed the highest nucleotide identity to EYVV-Fz1 (GenBank accession NC_010307.1), consistent with the small RNA sequencing results, indicating that it is an isolate of EYVV, which was designated as EYVV-Gx, with ‘Gx’ referring to its origin in Guangxi. While we cannot completely rule out the presence of other viruses at extremely low levels, these results indicate that EYVV-Gx is the primary virus responsible for the observed symptoms.

### 3.2 PacBio SMRT sequencing and transcriptome assembly of *E. sonchifolia*

PacBio SMRT sequencing was performed to generate a reference transcriptome of *E. sonchifolia*. The workflow for PacBio full-length transcriptome sequencing and downstream bioinformatic analyses in *E. sonchifolia* is shown in **Supplementary Figure 2**. A total of 46.47 GB of clean data, comprising 501,210 raw polymerase reads, was obtained from all ten samples. After removing adaptor sequences and low-quality reads, 26,003,005 subreads (45.31 GB) were generated. Using the SMRT Portal RS_IsoSeq protocol, 375,663 CCS were obtained, of which 233,827 were identified as FLNC reads containing poly (A) tails, with an average length of 1,802 bp.

Alternative splicing of pre-mRNA enables the generation of multiple splice isoforms from a single gene (Nilsen and Graveley, 2010). To remove redundant isoforms from the FLNC reads, ICE analysis was applied to classify and cluster the reads. Each resulting cluster was considered to represent a unique isoform. The resulting isoforms were further polished, yielding 23,398 high-quality transcripts with an average length of 1,833 bp.

Due to the relatively high error rate of SMRT sequencing (approximately 12-15%), consensus isoforms were further corrected using LoRDEC (Salmela and Rivals, 2014) with high-quality short reads. To reduce sequence redundancy, clustering analysis was performed using the CD-HIT program (Fu et al., 2012). Ultimately, 19,525 non-redundant consensus isoforms were obtained and used as the reference transcriptome for short-read mapping. An overview of the SMRT results in *E. sonchifolia* is provided in **Supplementary Table 2**. The lengths of the consensus isoforms ranged from 56 bp to 9,428 bp, with an average length of 1,829 bp. The length distribution of the consensus isoforms is shown in **Supplementary Figure 3A**. Details of isoform clustering based on transcript number are provided in **Supplementary Figure 3B**.

### 3.3 Functional annotation and pathway analysis of *E. Sonchifolia* transcripts

Functional annotation of the 19,525 unigenes was performed using BLASTP searches against several public databases, including NR, Swiss-Prot, eggNOG, GO, TrEMBL, KEGG, and Pfam (E value < 1e-5). A total of 18,803 unigenes (96.3% of the total transcripts) were successfully annotated in at least one database. A total of 10,271 isoforms were annotated by five databases (GO, NR, eggNOG, KEGG, and Swiss-Prot). The highest proportion of unigenes (96.05%) was matched in the TrEMBL database, followed by 96.04% matched in the NR database. In total, 18,539 unigenes (94.95%) were annotated in eggNOG, 17,703 (90.67%) in GO, 15,698 (80.40%) in Swiss-Prot, and 17,160 (87.89%) in Pfam. However, only 11,094 unigenes were annotated in the KEGG database. Homology analysis showed that the largest proportion of *E. sonchifolia* transcripts (24.04%) was most similar to sequences from *Cynara cardunculus* var. *scolymus*, followed by *Helianthus annuus* (20.63%) and *Artemisia annua* (20.48%).

A total of 17,703 genes were assigned to 5,973 GO terms, which were further classified into three main GO categories: biological processes (BP; 3,530 terms), cellular components (CC; 711 terms), and molecular functions (MF; 1,732 terms). The most represented terms in the BP category were ‘biological process’ (9,686), ‘cellular process’ (8,324), and ‘metabolic process’ (7,138), reflecting the broad functional annotation of the transcriptome. The dominant terms in the CC category were ‘cellular component’ (10,514), ‘cellular anatomical entity’ (10,335), and ‘intracellular anatomical structure’ (6,950), indicating general subcellular localization annotations. In the MF category, genes annotated with the term ‘molecular function’ (14,101) were the most abundant, followed by ‘binding’ (9,194), and ‘catalytic activity’ (9,093), which suggests that a large proportion of genes encode proteins involved in molecular interactions and enzymatic functions. A total of 11,094 unigenes were assigned to 294 pathways in the KEGG database. The five most enriched KEGG pathways were: ‘Carbon metabolism (786)’, ‘Photosynthesis’ (562), ‘Oxidative phosphorylation’ (445), ‘Biosynthesis of amino acids’ (492), and ‘Ribosome’ (430). These pathways represent conserved core biological processes involved in energy metabolism, biosynthesis, and protein translation in plants.

### 3.4 Identification and characterization of CDSs, lncRNAs and SSRs from SMRT-Seq data

**Figure 2:**
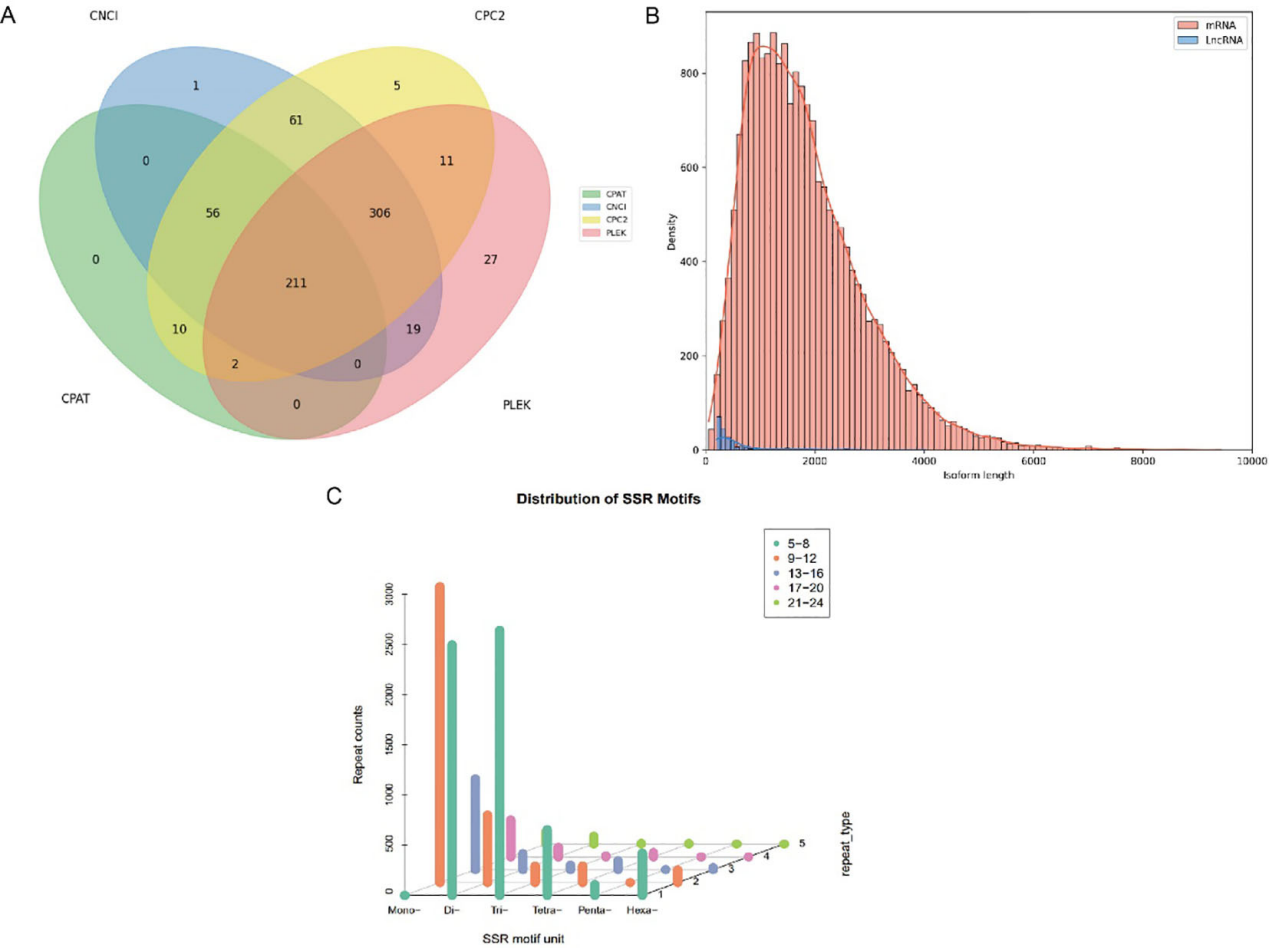
Long non-coding RNA (lncRNA) prediction, isoform length distribution, and simple sequence repeat (SSR) motif types in the *Emilia sonchifolia* (*E. sonchifolia*) transcriptome. **(A)** Venn diagram summarizing lncRNA predictions from CNCI, CPC2, CPAT, and PLEK analyses. **(B)** Density plot showing the length distribution of mRNA and lncRNA transcripts. **(C)** Distribution of SSR motif types identified in the transcriptome. All data were derived from PacBio long-read RNA sequencing of *E. sonchifolia.*.

TransDecoder software was used to predict putative CDSs from the assembled ISO-seq transcripts. Among the 19,525 ISO-seq transcripts, 16,986 transcripts containing complete open reading frames (ORFs) were identified as CDSs, with an average length of 1,415.07 bp. LncRNAs are defined as RNA molecules longer than 200 nucleotides that do not encode proteins, or that encode small peptides of fewer than 100 amino acids. LncRNAs regulate gene expression through transcriptional, post-transcriptional, and epigenetic mechanisms (Palos et al., 2023). In this study, four computational tools were employed to predict non-coding isoforms. A Venn diagram was generated to summarize lncRNA predictions from the four methods (**Figure 2A**). The numbers of non-coding isoforms predicted by CNCI, CPC2, PLEK, and CPAT were 654, 662, 576, and 279, respectively. Isoforms predicted as non-coding by all four methods were classified as lncRNAs, resulting in the identification of 211 high-confidence lncRNAs. The length distribution of isoforms was compared between mRNAs and lncRNAs. The results indicated that most lncRNAs were concentrated around 200 nucleotides in length (**Figure 2B**).

SSRs, also known as microsatellites, are short tandem repeats composed of mono-, di-, tri-, tetra-, penta-, and hexa-nucleotide motifs. SSRs are widely distributed in eukaryotic genomes and are commonly used as genetic markers to assess genetic variation (Srivastava et al., 2019). The MISA tool was used to detect SSRs. A total of 12,351 SSRs were identified, comprising six types of repeats. The distribution of SSR motifs is shown in **Figure 2C**. The most abundant SSR type was mono-nucleotide repeats (4,347), followed by di-nucleotide repeats (3,500). The numbers of tri-, tetra-, penta-, and hexa-nucleotide repeats were 2,867, 958, 111, and 568, respectively.

### 3.5 Differential gene expression analysis between Mock and Vln *E. sonchifolia* plants using Illumina RNA-seq

DEGs between the Mock and Vln plants were identified using Illumina RNA-Seq. Five biological replicates each type was used for library construction. A total of 64.6 Gb of clean reads were obtained from all samples, with over 94% of reads having a Q30 quality score (>99.9%). An overview of the Illumina HiSeq sequencing data for *E. sonchifolia*, including five plants per type, is provided in **Supplementary Table 3**. All Illumina clean reads were mapped to the SMRT full-length transcriptome using Bowtie2, with an average mapping ratio of 71.85% across the ten samples. This indicated that the SMRT-derived transcriptome captured the majority of the genetic information in *E. sonchifolia*. Across all samples, 20.96%-23.73% of reads were uniquely mapped, while 46.12%-52.23% were mapped to multiple locations.

**Figure 3:**
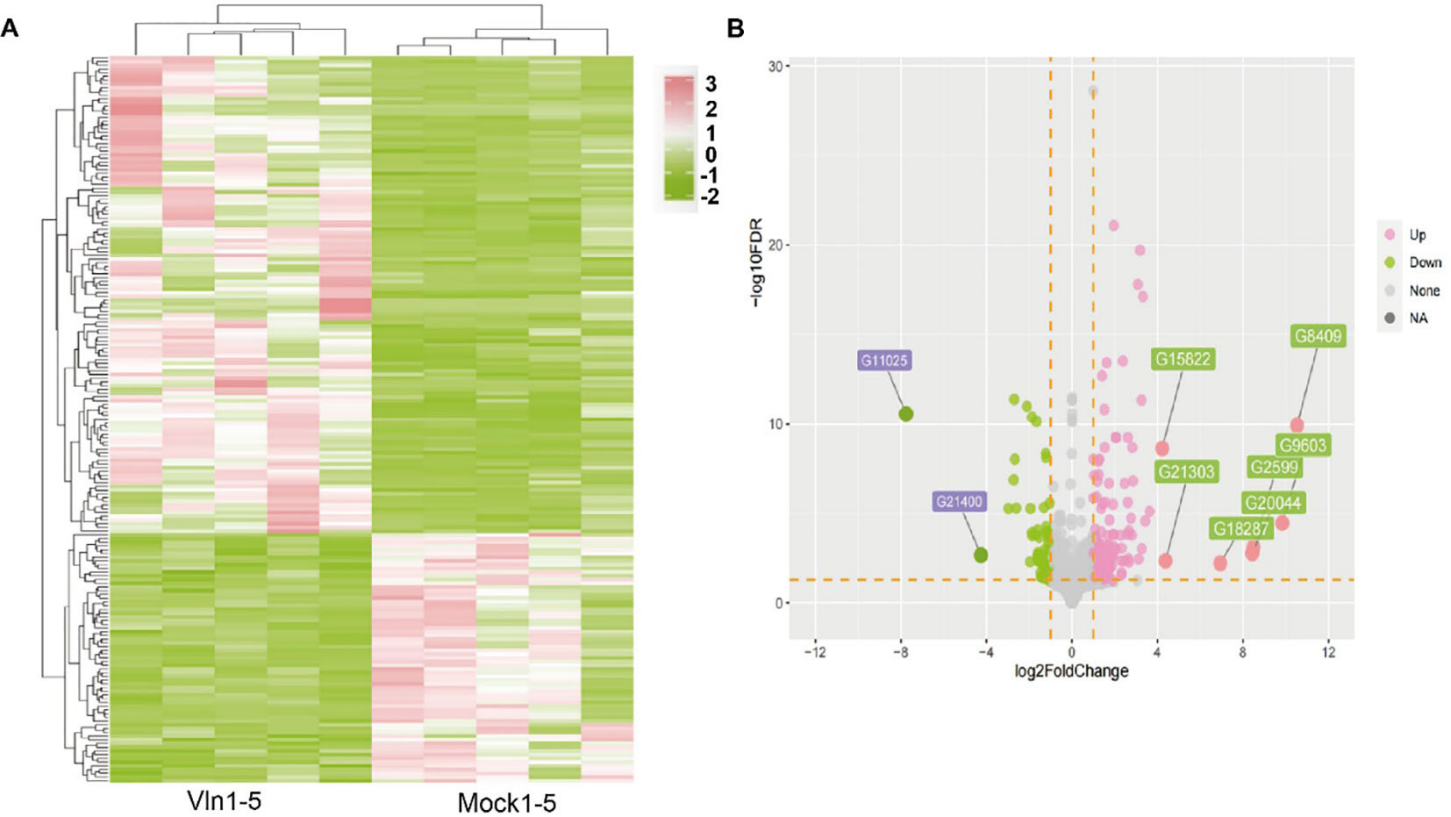
Differential gene expression analysis between symptomatic (Vln) and asymptomatic (Mock) *Emilia sonchifolia* (*E. sonchifolia*) leaf samples **(A)** Heatmap showing the relative expression levels of differentially expressed genes (DEGs) across all 10 *E. sonchifolia* leaf samples, including five Vln samples (Vln1-Vln5) and five Mock samples (Mock1-Mock5). Hierarchical clustering of samples and genes was performed using Euclidean distance and complete linkage, with expression values normalized as Z-scores. Colors indicate normalized expression levels (Z-scores of FPKM values). **(B)** Volcano plot illustrating the distribution and overall expression patterns of DEGs between the Vln and Mock groups. The x-axis represents log_2_ (fold change of Vln/Mock lines), and the y-axis represents the –log_10_(FDR). Red dots indicate upregulated DEGs, and green dots indicate downregulated DEGs, with significance thresholds of |log_2_ fold change| > 1 and FDR < 0.05. A total of 195 genes were identified as significantly differentially expressed. Statistical analysis: Differential expression analysis was performed using DESeq2 (version 1.18.1), with significance thresholds as indicated.

In total, 195 DEGs were identified between Mock and Vln plants. Among these, 128 genes were significantly upregulated in the Vln plants, whereas 67 genes were significantly downregulated in Vln plants, based on a log_2_ (Vln/Mock) fold-change threshold of ±1. A heatmap (**Figure 3A**) and a volcano plot (**Figure 3B**) were generated to visualize normalized DEGs expression across the ten samples. Samples from the same treatment group clustered together, indicating that genes in these samples exhibited similar relative expression patterns.

### 3.6 GO enrichment and KEGG pathway analysis of DEGs

**Figure 4:**
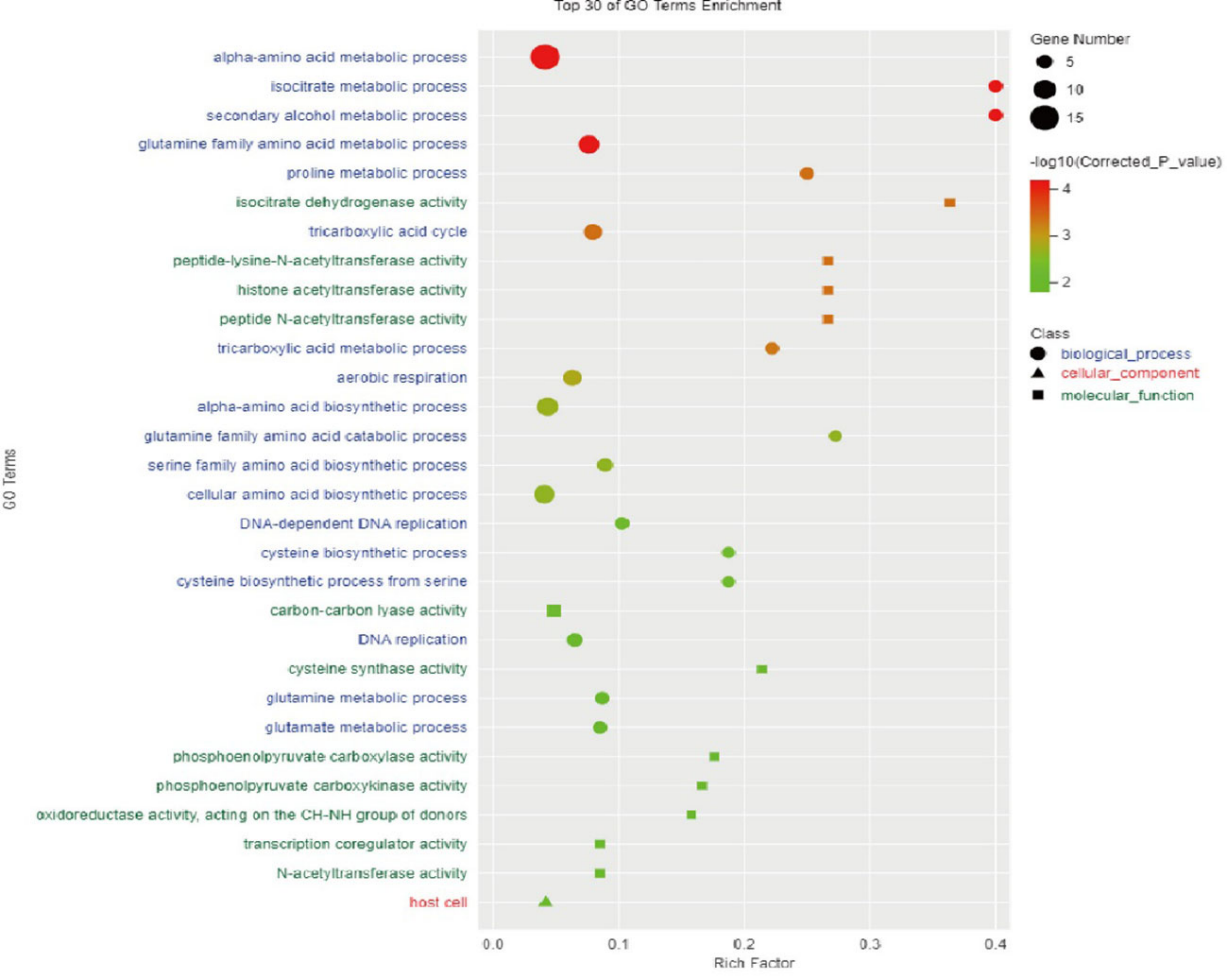
Bubble chart of the top 30 significantly enriched Gene Ontology (GO) terms. GO terms are grouped into three categories: biological process (BP), molecular function (MF), and cellular component (CC). The rich factor represents the ratio of differentially expressed genes (DEGs) associated with a specific GO term to the total number of background genes annotated for that term. The size of each bubble indicates the number of DEGs associated with the corresponding GO term, and the color gradient (–log_10_ adjusted *p*-value) reflects the significance of enrichment. Statistical significance was determined based on an adjusted *p*-value (padj) threshold of < 0.05. GO pathway enrichment analyze was conducted using the clusterProfiler R package.

**Figure 5:**
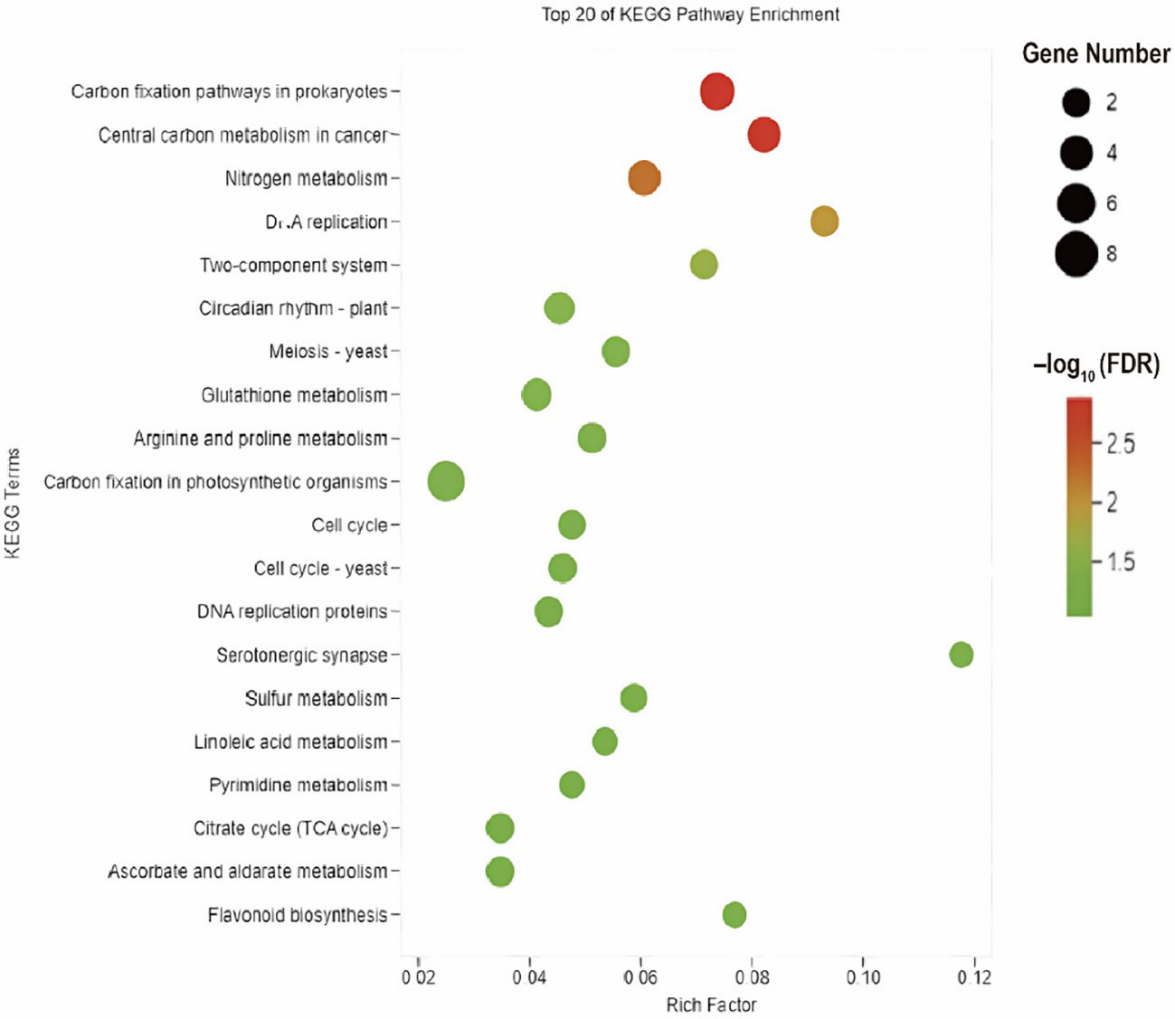
Top 20 significantly enriched Kyoto Encyclopedia of Genes and Genomes (KEGG) pathways (bubble chart). The rich factor represents the ratio of differentially expressed genes (DEGs) associated with a specific pathway to the total number of background genes annotated for that pathway. The size of each bubble indicates the number of DEGs associated with the pathway, and the color gradient (–log_10_ adjusted *p*-value) reflects the significance of enrichment. Statistical significance was determined based on an adjusted *p*-value (padj) threshold of < 0.05. KEGG pathway enrichment analyze was conducted using the clusterProfiler R package.

GO enrichment analysis was conducted to classify the DEGs according to their predicted functional roles. The 195 DEGs were associated with 458 functional groups across the three main GO categories: BP (230), CC (53), and MF (175). Twenty-eight of the 230 GO terms were significantly enriched in the BP category (adjusted *p*-value < 0.05), with the top three terms being ‘alpha-amino acid metabolic process’, ‘glutamine family amino acid metabolic process’, and ‘isocitrate metabolic process’ (**Figure 4**). In the CC category, eight GO terms were significantly enriched, with the top three terms related to ‘host cellular component’, ‘host cell part’, and ‘host intracellular part’ (**Figure 4**). In the MF category, 27 GO terms were significantly enriched, with ‘isocitrate dehydrogenase activity’, ‘histone acetyltransferase activity’, ‘peptide N-acetyltransferase activity’ ranking as the top three based on adjusted *p*-value (**Figure 4**). The 30 most enriched GO terms, ranked by enrichment factor, are shown in **Figure 4**. KEGG pathway analysis assigned the DEGs to 93 pathways. The top 20 enriched KEGG pathways, ranked by enrichment factor, are presented in **Figure 5**. Among these, 12 pathways were significantly enriched (adjusted *p*-value < 0.05), including ‘carbon fixation pathways in prokaryotes’, ‘central carbon metabolism in cancer’, ‘nitrogen metabolism’, ‘DNA replication’, ‘two-component system’, ‘circadian rhythm – plant’, ‘meiosis – yeast’, ‘glutathione metabolism’, ‘arginine and proline metabolism’, ‘carbon fixation in photosynthetic organisms’, ‘cell cycle’ and ‘cell cycle – yeast’.

### 3.7 Defense response pathways in the Vln plants: insights from differential gene expression

Based on well-established plant defense–related pathways, DEGs were further classified into several functional subgroups (**Supplementary Table 4**), including cell wall metabolism, transcriptional regulation, hormone signaling, ROS metabolism, DNA replication, and pathogenesis. As shown in **Supplementary Table 4**, upregulated genes related to cell wall modification and ROS metabolism exhibited significantly higher expression in the Vln plants, whereas downregulated genes, such as several auxin-responsive genes, displayed the opposite expression pattern. Among pattern-triggered immunity (PTI)-associated defense responses, cell wall remodeling and ROS scavenging play crucial roles. In this study, 12 DEGs involved in ROS metabolism were identified, with 10 genes significantly upregulated in the Vln plants and 2 genes significantly downregulated in the Vln plants. Representative enzymes included isocitrate dehydrogenases, monodehydroascorbate reductase, L-ascorbate oxidase, glutaredoxin, and proline dehydrogenase. Additionally, upregulated genes associated with cell wall remodeling included genes encoding pectinesterase, expansin, alpha/beta hydrolase fold protein, alpha-galactosidase, pyruvate kinase, and fructose-bisphosphate aldolase. In this study, a total of 88 TF-related DEGs were identified, belonging to 17 TF families (**Supplementary Figure 4**). Based on abundance, the C3H and MYB-related families had the highest number of isoforms, with 8 each. Additionally, the Ethylene Response Factor (ERF), Far-Red Impaired Response 1 (FAR1), and Auxin Response Factor (ARF) families also showed a relatively high number of differentially expressed TFs, with 6 to 7 TFs each (**Supplementary Figure 4**). These data suggest that viral infection appears to regulate a broad range of TF families, influencing the plant’s transcriptional response. Notably, four TFs from the Broad-Complex, Tramtrack, and Bric-à-brac/POxvirus and Zinc finger (BTB/POZ) and Transcriptional co-activator with PDZ-binding motif (TAZ) families exhibited significant upregulation in response to viral infection, showing greater expression changes compared to other families. Additionally, two plant-specific members of the RWP-RK TF family and one nuclear TF Y were downregulated following infection. These TFs were found to be differentially expressed when comparing infected Vln plants to mock-treated controls.

Several upregulated DEGs annotated as leucine-rich repeat-containing receptors (NLRs) were significantly induced in the Vln plants, suggesting the involvement of intracellular immune signaling pathways during viral infection. Furthermore, DEGs associated with abscisic acid (ABA) and auxin signaling pathways, as well as virus DNA replication and energy metabolism, were also identified, with some genes being upregulated and others downregulated in the Vln plants.

### 3.8 Validation of candidate gene expression by RT-qPCR

**Figure 6:**
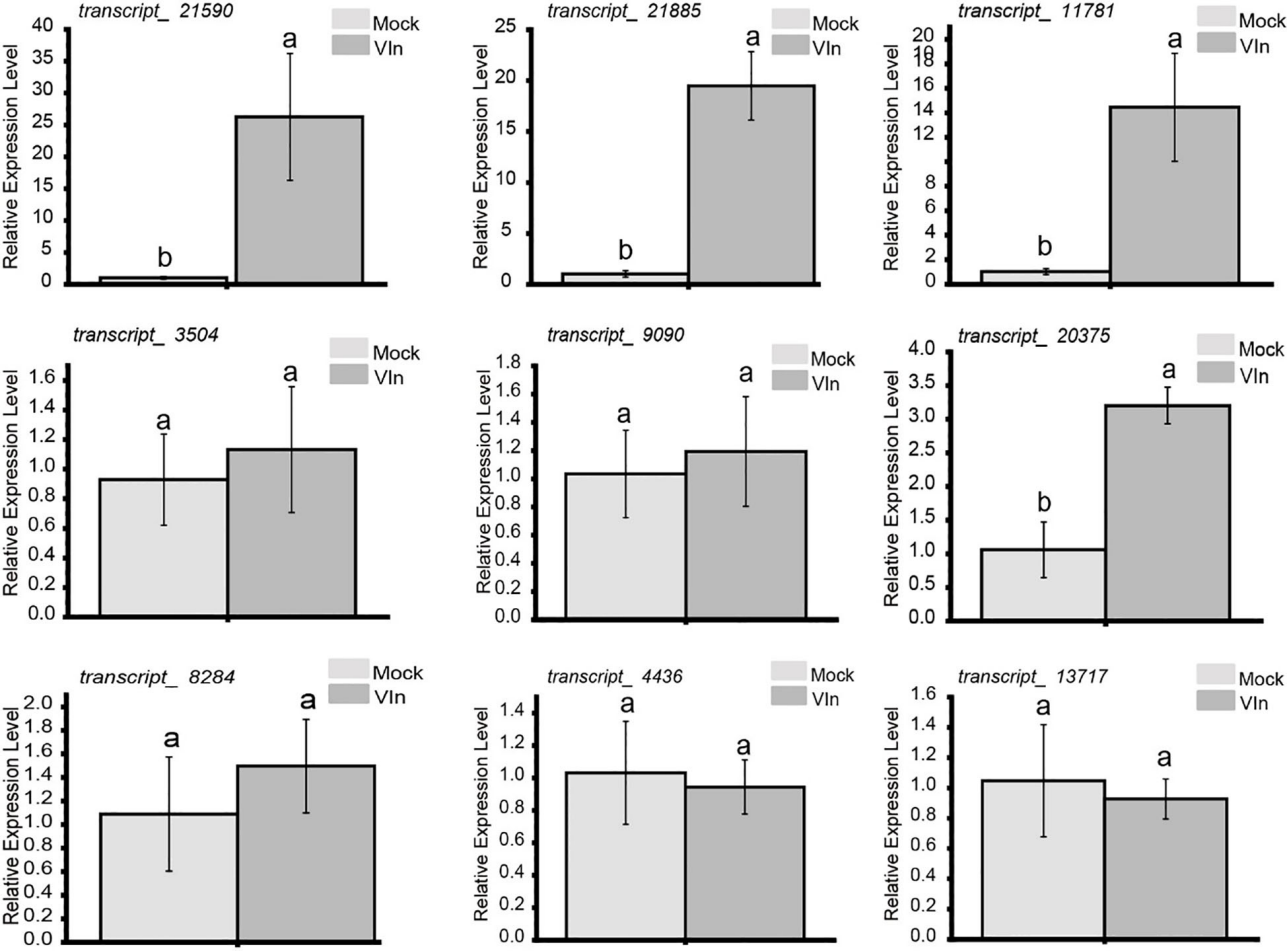
Expression profiles of selected genes in leaf tissue of Mock and Vln plants. Relative expression levels were determined by reverse transcription quantitative polymerase chain reaction (RT-qPCR) and are presented as fold changes relative to those in Mock plants. Gene expression was quantified using the 2^-ΔΔCt^ method, with *glyceraldehyde-3-phosphate dehydrogenase* (*GAPDH*) as the internal reference gene for data normalization. Error bars represent the mean ± SEM. RT-qPCR analyses were conducted using five independent biological replicates, with three technical replicates performed for each biological sample. Statistical significance between Mock and Vln plants was determined using Student’s t-test; different letters indicate significant differences at *p* < 0.05. Transcript annotations are as follows: *transcript_21590*, glycine-rich protein; *transcript_21885*, glutaredoxin; *transcript_11781*, alpha/beta hydrolases fold protein; *transcript_3504*, plant lipoxygenase; *transcript_9090*, ferredoxin–nitrite reductase; *transcript_20375*, major latex protein; *transcript_8284*, proline dehydrogenase; *transcript_4436*, DNA replication licensing factor minichromosome maintenance complex component 4 (MCM4); and *transcript_13717*, BTB/POZ and TAZ transcription factor.

**Figure 7:**
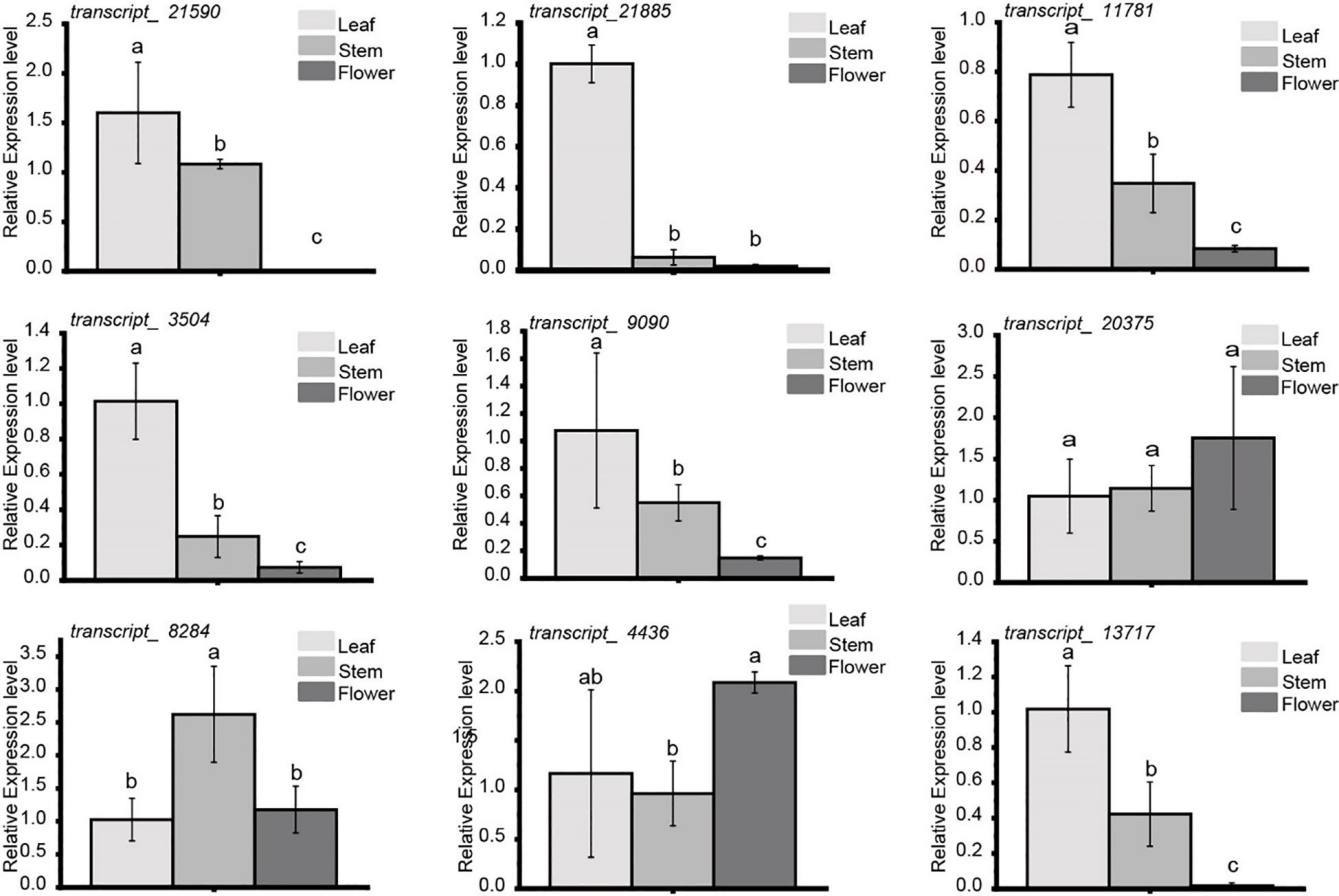
Expression profiles of nine selected genes across three different tissues (leaf, stem, and flower) of Vln plants. Relative expression levels were determined by reverse transcription quantitative polymerase chain reaction (RT-qPCR) and are presented as fold changes relative to leaf tissue. Gene expression was quantified using the 2^-ΔΔCt^ method, with *glyceraldehyde-3-phosphate dehydrogenase* (*GAPDH*) as the internal reference gene for data normalization. Error bars represent the mean ± SEM. RT-qPCR analyses were conducted using three independent biological replicates, with three technical replicates performed for each biological sample. Statistical significance among tissues was determined by one-way ANOVA followed by Tukey’s *post-hoc* test; different letters indicate significant differences at *p* < 0.05. Transcript annotations are as follows: *transcript_21590*, glycine-rich protein; *transcript_21885*, glutaredoxin; *transcript_11781*, alpha/beta hydrolase fold protein; *transcript_3504*, plant lipoxygenase; *transcript_9090*, ferredoxin–nitrite reductase; *transcript_20375*, major latex protein; *transcript_8284*, proline dehydrogenase; *transcript_4436*, DNA replication licensing factor MCM4; and *transcript_13717*, BTB/POZ and TAZ TF.

To validate the accuracy of the RNA-seq results, nine DEGs were selected for RT-qPCR analysis to compare their expression levels between different groups. These genes are associated with various defense responses, including cell wall remodeling, transcriptional regulation, ROS metabolism, and hormone signaling. Gene IDs and the corresponding annotated proteins are listed in **Supplementary Table 4**. As shown in **Figure 6**, several DEGs showed significant expression differences between Mock and Vln plants, consistent with the RNA-seq data. Although some genes did not reach statistical significance, similar expression trends were still observed. qRT-PCR validation of nine selected DEGs further supported the RNA-seq results, indicating overall consistency between the two methods (**Supplementary Figure 5**). The mean Ct values and corresponding standard deviations for the selected genes across all biological replicates are provided in **Supplementary Tables 5**, **6**. The differences may be attributed to variations in sensitivity between RT-qPCR and RNA-seq. Nevertheless, the overall results indicate a high level of reliability of the RNA-seq analysis. The expression patterns of these genes were further examined in different tissues—leaf, stem, and flower—of the Vln plants, revealing distinct tissue-specific regulation (**Figure 7**). Genes encoding cell wall-related proteins, such as hydrolase and glycine-rich protein, were predominantly expressed in leaves and stems. The plant cell wall plays a crucial role in defense response by serving as a physical barrier against pathogen invasion (Wolf, 2022). Thus, the induction of cell wall-related genes in leaf tissue is consistent with a possible role for these enzymes in structural reinforcement during virus-induced defense responses. ROS-related enzymes are primarily involved in PTI. Among the DEGs, several genes encoded ROS-related enzymes, including glutaredoxin, monodehydroascorbate reductase, and ferredoxin–nitrite reductase. For example, RT-qPCR results showed that a *glutaredoxin* gene was more highly expressed in leaves than in other tissues. This observation implies that leaf tissue may be the primary site of viral exposure, based on the higher expression levels of ROS-related genes.

In contrast, genes that were highly expressed in flower tissue were mainly associated with hormonal and metabolic regulation. Representative DEGs included a gene encoding a major latex protein, which is often associated with salicylic acid (SA)-induced defense, and a gene encoding a DNA replication licensing factor. This suggests that these genes may contribute to reproductive development and potentially in defense responses. Additionally, genes encoding TFs, such as BTB/POZ and TAZ TFs, also showed tissue-specific expression, with higher expression levels in leaves compared to other tissues. These TFs may be involved in effector-triggered immunity (ETI), contributing to the activation of more robust and sustained defense responses. Altogether, the expression analysis demonstrates that the regulation of defense-related genes dependent not only on genotype but is also tightly controlled in a tissue-specific manner.

## 4 Discussion

To date, numerous studies on begomovirus–plant interactions have revealed transcriptional changes associated with virus infection (He et al., 2024a, He et al., 2024b, He et al., 2024c). However, information regarding the medicinal plant *E. sonchifolia* remains limited. In this study, transcriptomic profiling combining SMRT sequencing with Illumina sequencing was conducted to reveal virus-responsive transcriptional reprogramming in *E. sonchifolia*. Our results suggest complex transcriptional changes underlying the *E. sonchifolia–*EYVV interaction. In total, 195 DEGs were identified and classified into key pathways involved in stress and immune-related processes. The enrichment of genes associated with stress response, redox regulation, and cell wall remodeling suggests that viral infection is closely associated with the observed transcriptional changes.

PTI and ETI represent interconnected layers of plant immune responses that may be associated with transcriptional changes observed in this study. ROS generation is a hallmark of plant defense responses against biotic stress (Wu et al., 2023). In this study, several ROS-scavenging genes were significantly upregulated in the Vln plants, including monodehydroascorbate reductase (MDHAR) and glutaredoxin genes, suggesting that ROS homeostasis is critical in the plant’s response to viral infection. However, excessive ROS accumulation can cause cellular damage, and plants therefore activate antioxidant systems to maintain redox balance. Previous studies have shown that enzymes with antioxidant activities play important roles in ROS detoxification (Hasanuzzaman et al., 2019; Johnston et al., 2015). The upregulation of *isocitrate dehydrogenase* (*IDH*) genes further supports the involvement of ROS in the plant’s defense, as *IDH* manages ROS levels indirectly by providing reducing power for antioxidant enzymes. ROS may also act as a signaling molecule, triggering downstream immune responses, including the activation of auxin signaling, which is known to modulate immune responses in plants (Haghpanah et al., 2025). The upregulation of ROS-scavenging genes and auxin-responsive genes suggests a potential interaction between ROS and auxin signaling pathways, which warrants further investigation.

The plant cell wall serves as both a physical barrier and an active component of plant immunity (Bacete et al., 2018). In this study, several cell wall-related genes were differentially expressed. A gene encoding extensin was significantly induced by viral infection. Cross-linking of extensins has been associated with strengthening of the cell wall during pathogen challenge (Castilleux et al., 2021). Additionally, two *pectinesterase* genes were significantly upregulated, suggesting that changes in pectin methyl-esterification may be associated with altered cell wall properties during viral infection.

Epigenetic regulation may contribute to transcriptional reprogramming during infection. In this study, four *histone acetyltransferase-related* genes were identified as differentially expressed in the Vln plants, suggesting a potential role of epigenetic regulation in virus-responsive transcriptional changes.

Several DEGs related to DNA replication and cell cycle regulation were also identified. Notably, genes encoding replication licensing factors such as MCM4 and MCM3 homologs were significantly upregulated in Vln plants. Since geminiviruses rely on host DNA replication machinery for genome replication (Shakir et al., 2023; Wu et al., 2023), these changes may reflect altered host DNA replication activity during infection.

Phytohormone-related genes also showed differential expression upon viral infection. For example, *small auxin up RNA* (*SAUR*) genes were significantly downregulated in the Vln plants. *SAURs*, the largest family of early auxin response genes, have been reported to be associated with virus-induced growth and symptom development (Geng et al., 2017).

The downregulation of *SAUR* genes in the Vln plants suggests that auxin responses might be reduced as part of the plant’s strategy to limit growth and prioritize defense mechanisms. While *SAUR* genes are involved in auxin signaling, which can activate immune responses, their downregulation might help to restrict growth and minimize symptom development (Bao et al., 2024). This could reflect a trade-off between defense activation and growth regulation during viral stress. In contrast, an ABA-related gene was upregulated in Vln plants. ABA has been shown to modulate plant immune responses by regulating callose deposition and gene expression, which are crucial for limiting pathogen entry and controlling disease progression (Alazem and Lin, 2017; Chi et al., 2025). The upregulation of ABA-related genes is associated with the plant’s response to viral infection, and further studies are needed to determine if ABA activation of stress-related pathways plays a role in reducing viral replication or facilitating systemic acquired resistance.

In this study, both the C3H and MYB-related TF families were prominently represented, with eight DEGs identified in each family. Additionally, four *MYB* genes were also identified. These families are known for their roles in regulating plant responses to stress and immunity by modulating gene expression, hormone signaling and growth-related pathways (Feng et al., 2025; Lu et al., 2025). The differential expression of these TFs highlights their potential involvement in the plant’s transcriptional response to begomovirus infection. Four differentially expressed TFs were identified as containing both BTB/POZ and TAZ domains. Given the presence of these domains, these TFs may potentially belong to either the BTB/POZ or TAZ families, or possibly represent a cross-functional type that spans both families. In plant viral defense, BTB/POZ and TAZ domains primarily function through the regulation of SA signaling and the ubiquitin-proteasome system (Zhou et al., 2022), and their upregulation suggests these TFs may play a role in modulating the plant’s transcriptional response to viral infection. The observed downregulation of two *RWP-RK* genes in the Vln plants likely reflects a programmed growth-defense trade-off. By suppressing these key regulators which are known to regulate nitrogen assimilation, the plant may reallocate metabolic resources toward antiviral signaling (Du et al., 2025).

These interpretations are based on transcriptomic evidence and functional annotation, and experimental validation will be required to confirm the roles of these pathways in EYVV–host interactions. A limitation of this study is the absence of artificial inoculation experiments and independent infection assays, which prevents definitive causal attribution between viral infection and observed transcriptional changes. While our transcriptomic data suggest associations between various pathways and viral infection, causal relationships between these factors remain speculative, and future studies with controlled infection systems are necessary to confirm these findings. In addition, as Vln and Mock plants were collected from a field population in a single season, variation in viral load, genotype, microenvironment, and developmental stage may also contribute to transcriptomic differences. Therefore, the results should be interpreted as field-based infection-associated responses rather than experimentally validated causal effects.

In addition, the PacBio reference transcriptome was constructed using pooled samples from all ten plants. While this approach facilitates identification of full-length transcripts and isoforms, it may reduce sensitivity for treatment-specific or low-abundance transcripts. Multi-mapping of Illumina reads may also be influenced by transcript redundancy and isoform similarity. Although probabilistic assignment was performed using RSEM, some ambiguity in expression quantification may remain. Small RNA sequencing was used to detect virus-derived siRNAs, together with RT-PCR and Sanger sequencing for validation; However, this approach cannot distinguish active viral replication from environmental contamination or latent infections, and the presence of low-abundance or co-infecting viruses cannot be excluded. In our analysis, we initially observed a weak correlation between the RNA-seq and RT-qPCR results. However, after excluding a few data points (specifically *transcript*_8284, *transcript*_4436, and *transcript*_13717) that showed large discrepancies, the Pearson correlation coefficient improved to 0.747, indicating a moderate to strong positive correlation between the two methods. While some differences remain, likely due to the higher sensitivity of RT-qPCR for low-abundance transcripts, as well as other potential technical factors such as sample preparation or assay-specific variations, both methods still demonstrated strong consistency overall. These findings suggest that RNA-seq and RT-qPCR provide complementary and reliable insights into gene expression changes in *E. sonchifolia*, with each method contributing valuable data.

Taken together, these limitations should be considered when interpreting the results. Future studies using controlled infection systems, multi-season sampling, and functional validation will be necessary to confirm the molecular mechanisms underlying EYVV–host interactions in *E. sonchifolia*.

## 5 Conclusion

This study generated the first transcriptome-wide dataset of *E. sonchifolia* under natural geminivirus infection and identified key gene expression changes associated with viral infection. DEGs were identified and were predominantly involved in cell wall modification, ROS scavenging, epigenetic regulation, and hormone signaling pathways (ABA and auxin). In addition to characterizing infection-associated transcriptional reprogramming, this work provides a high-quality transcriptomic resource for *E. sonchifolia*, contributing foundational data for future functional studies on virus–host interactions and molecular improvement of medicinal plants.

## Data Availability

The datasets presented in this study can be found in online repositories. The names of the repository/repositories and accession number(s) can be found below: https://www.ncbi.nlm.nih.gov/, PRJNA1107498.

## Supplementary material

Supplementary Table 1 Primers used for RT-qPCR and PCR amplification of the viral genome.

Supplementary Table 2 Overview of the single molecule real-time sequencing (SMRT) in *Emilia Sonchifolia*.

Supplementary Table 3 Overview of the Illumina HiSeq sequencing data for *Emilia Sonchifolia*, including five asymptomatic plants (Mock1-5) and five symptomatic plants (Vln1-5).

Supplementary Table 4 Representative differentially expressed genes (DEGs) identified between the asymptomatic plants (designated as Mock) and virus-infected plants (designated as Vln).

Supplementary Table 5 Mean cycle threshold (Ct) values and standard deviations (± SD) of nine selected differentially expressed genes (DEGs) and the reference gene across all biological replicates in the Mock and Vln *Emilia sonchifolia* plants.

Supplementary Table 6 Mean cycle threshold (Ct) values and standard deviations (± SD) of nine selected differentially expressed genes (DEGs) and the reference gene across all biological replicates in leaf, stem, and flower tissues of Vln *Emilia sonchifolia* plants.

Supplementary Figure 1 Representative agarose gel electrophoresis of EYVV detection in symptomatic (Vln) and asymptomatic (Mock) plants.

Supplementary Figure 2 Workflow of PacBio full-length transcriptome sequencing and bioinformatic analysis in *Emilia sonchifolia*.

Supplementary Figure 3 Length distribution and clustering of consensus isoforms from Single-Molecule Real-Time (SMRT) sequencing.

Supplementary Figure 4 Differential expression of transcription factor (TF) families in response to virus infection in Vln plants.

Supplementary Figure 5 Comparison of RNA-seq and qRT-PCR log_2_ fold change values for nine selected differentially expressed gene (DEGs).
