# Supplementary material for: Integrated transcriptomic analysis reveals transcriptional changes associated with begomovirus infection in the medicinal plant Emilia sonchifolia
Source: Front Plant Sci. 2026 May 26;17:1827690. doi: 10.3389/fpls.2026.1827690 (PMC13246636; doi:10.3389/fpls.2026.1827690)
Supplement: Supplementary Table 1 — Primers used for RT-qPCR and PCR amplification of the viral genome. [file DataSheet1.docx]

Supplementary Materials

**Table S1** Primers used for RT-qPCR and PCR amplification of the viral genome

| Gene ID | Sequence number | Forward primer/Reverse Primer | Purpose |
| --- | --- | --- | --- |
| GAPDH | cell_1_transcript_13030 | GAATGTCCTTCCGAGTCCCC/GACCTGCTGTCTCCGACAAA | qPCR, reference gene |
| G21590 | cell_1_transcript_21590 | AGTATGGTGGTGATGACCGTG/AATTCCCTCCGTGGTTCCTTC | qPCR |
| G4436 | cell_1_transcript_4436 | GAAGGGACAACATGGTGGAGT/TCACTTGCAAGCGTCCCAA | qPCR |
| G13717 | cell_1_transcript_13717 | ATTTGCACAGAAGGTTGCACG/ACAACCTCCACTAGCTCTCTTC | qPCR |
| G3504 | cell_1_transcript_3504 | GACCCGACTGATGAGGAATGG/CGCAGCTTGTATCTGAAGATTAGC | qPCR |
| G21885 | cell_1_transcript_21885 | GGCACTGTCTTCGATTGGGTA/CCCATAAAGCTCCAGCACTGA | qPCR |
| G8284 | cell_1_transcript_8284 | TATGTGGAGCGAAAGCCAGTT/GCAAGAATGAGACCACCAGGA | qPCR |
| G20375 | cell_1_transcript_20375 | GGCCAAGGAAGTGATTGAAGC/TTCTCTTCTCCGTGTGTGTCC | qPCR |
| G9090 | cell_1_transcript_9090 | TTACCCGTCCAGTACGAATGC/CCCAAGTAAACATCGGCTCCT | qPCR |
| G11781 | cell_1_transcript_11781 | TCGCCATACTTGAAAGACGAA/TGTTAACCCACCCTAAACTCCT | qPCR |
| Eyvv | isolate YL-1 | CAGCCGTCCATCAATACTTA/TTTCCTAACATACCCTCACTG | Viral genome PCR |
| Eyvvad | isolate YL-1 | ATGGGCTTGAATAGTAATGGG/CAAATTGAAGCCGTTAAGACC | Viral genome PCR |

**Table S2** Overview of the single molecule real-time sequencing (SMRT) in *Emilia Sonchifolia*

| Name | Polymerase reads | Subreads | CCS | FLNC | Consensus isoforms reads | High-quality  isoforms  reads |
| --- | --- | --- | --- | --- | --- | --- |
| Total_bases | 46.47Gbp | 45.31Gbp | 741,200,301bp | 421,141,227 | 42878575 | 35710319 |
| Total_number | 501,210 | 26,003,005 | 375,663 | 233,827 | 23398 | 19525 |
| Mean length (bp) | 92718 | 1742 | 1,974 | 1,802 | 1833.574 | 1829 |
| N50 (bp) | 170,596 | 1,917 | 2293 | 2160 | 2254 | 2263 |

**Table S****3**Overview of the Illumina HiSeq sequencing data for *Emilia Sonchifolia*, including five asymptomatic plants (Mock1-5) and five symptomatic plants (Vln1-5)

| Sample name | Raw_bases (G) | Clean_bases (G) | Clean_rate (%) | Q20 (%) | Q30 (%) | GC (%) |
| --- | --- | --- | --- | --- | --- | --- |
| Mock1 | 6.37 | 6.19 | 98.24 | 98.10 | 93.82 | 43.09 |
| Mock2 | 5.91 | 5.80 | 98.76 | 98.54 | 94.85 | 42.32 |
| Mock3 | 8.20 | 8.00 | 98.52 | 98.36 | 94.37 | 42.49 |
| Mock4 | 6.19 | 6.05 | 98.55 | 98.40 | 94.49 | 42.50 |
| Mock5 | 7.46 | 7.30 | 98.67 | 98.47 | 94.68 | 42.51 |
| Vln1 | 6.54 | 6.38 | 98.44 | 98.38 | 94.43 | 43.28 |
| Vln2 | 5.89 | 5.74 | 98.33 | 98.26 | 94.11 | 42.68 |
| Vln3 | 6.91 | 6.76 | 98.60 | 98.46 | 94.66 | 43.75 |
| Vln4 | 6.49 | 6.36 | 98.71 | 98.56 | 94.92 | 42.67 |
| Vln5 | 6.15 | 6.02 | 98.66 | 98.52 | 94.79 | 42.27 |

**Table S4.** Representative Differentially expressed genes (DEGs) identified between the asymptomatic plants (designated as Mock) and virus-infected plants (designated as Vln). Genes are grouped by functional class. Log₂ (fold change) values represent expression differences between the Vln plants and Mock Plants (Vln/Mock). Positive values indicate up-regulation in the Vln plants, whereas negative values indicate down-regulation in the Vln plants. Gene annotations are based on sequence similarity.

| Class | Gene ID | Log₂ (fold change) | Descriptin |
| --- | --- | --- | --- |
| Cell wall metabolism | cell_1_transcript_10659  cell_1_transcript_21590 | -1.04325  2.632142688 | fructose-bisphosphate aldolase  Glycine-rich protein-like |
|  | cell_1_transcript_11781 | 2.66259268 | alpha/Beta hydrolase fold protein |
|  | cell_1_transcript_17225 | 1.035349283 | Cell wall-associated hydrolase |
|  | cell_1_transcript_17664 | 2.822593278 | extensin |
|  | cell_1_transcript_21136 | 1.252366452 | Alpha-galactosidase |
|  | cell_1_transcript_9398 | 1.036812285 | Pectinesterase |
|  | cell_1_transcript_8344 | 1.39076086 | Cell wall-associated hydrolase |
|  | cell_1_transcript_9293 | 1.254682087 | Pyruvate kinase |
|  | cell_1_transcript_10257 | 1.158284009 | Pyruvate kinase |
|  | cell_1_transcript_9422 | 1.367840457 | Pectinesterase |
| TF | cell_1_transcript_1159 | -1.211978339 | The plant specific RWP-RK TF |
|  | cell_1_transcript_13107 | -1.595959779 | nuclear transcription factor Y |
|  | cell_1_transcript_13663 | 1.630136348 | BTB/POZ and TAZ TF |
|  | cell_1_transcript_13717 | 1.936029362 | BTB/POZ and TAZ TF |
|  | cell_1_transcript_14738 | 2.06294536 | BTB/POZ and TAZ TF |
|  | cell_1_transcript_14856 | 1.762863108 | BTB/POZ and TAZ TF |
|  | cell_1_transcript_3203 | -2.11068452 | Putative plant regulator RWP-RK family protein |
| Pathogenesis-related | cell_1_transcript_15389 | -1.023732569 | leucine-rich repeat receptor-like protein kinase |
|  | cell_1_transcript_3801 | 1.016314179 | leucine-rich repeat receptor-like protein kinase |
|  | cell_1_transcript_20375 | 1.314771808 | Major latex (Salicylic acid-inducible) |
| Auxin | cell_1_transcript_18083 | -1.863987556 | Auxin-responsive protein SAUR32 |
|  | cell_1_transcript_18940 | -1.056496392 | Auxin-responsive protein SAUR32 |
|  | cell_1_transcript_19899 | -1.56963975 | Auxin-responsive protein SAUR32 |
|  | cell_1_transcript_19938 | -1.477423013 | Auxin-responsive protein SAUR32 |
| ABA | cell_1_transcript_21254 | 3.424577833 | abscisic acid and environmental stress-inducible protein |
| ROS | cell_1_transcript_9975 | 1.069970115 | L-ascorbate oxidase |
|  | cell_1_transcript_4175 | 1.114758409 | Ribonucleoside-diphosphate reductase |
|  | cell_1_transcript_11671 | 1.957763543 | Isocitrate dehydrogenase |
|  | cell_1_transcript_12727 | 1.527610629 | Isocitrate dehydrogenase |
|  | cell_1_transcript_17461 | 1.704855447 | Isocitrate dehydrogenase |
|  | cell_1_transcript_18039 | 1.619944082 | Isocitrate dehydrogenase |
|  | cell_1_transcript_21885 | 1.937056747 | Glutaredoxin |
|  | cell_1_transcript_21611 | -1.334896175 | Glutaredoxin |
|  | cell_1_transcript_8046 | -1.022445291 | GDP-L-galactose phosphorylase |
|  | cell_1_transcript_8964 | 2.073406689 | Monodehydroascorbate reductase |
|  | cell_1_transcript_9086 | 2.035803137 | Monodehydroascorbate reductase |
|  | cell_1_transcript_8072 | 1.18218939 | Monodehydroascorbate reductase |
|  | cell_1_transcript_8284 | -2.728720542 | Proline dehydrogenase |
|  | cell_1_transcript_9090 | 1.710913541 | Ferredoxin--nitrite reductase |
|  | cell_1_transcript_3504 | 1.677582604 | Plant lipoxygenase |
| DNA replication | cell_1_transcript_4436 | 1.371639079 | DNA replication licensing factor MCM4 |
|  | cell_1_transcript_5038 | 1.965786514 | DNA replication licensing factor MCM3 |
|  | cell_1_transcript_14737 | 1.924720292 | DNA replication licensing factor MCM2 |

**Table S5.** Mean cycle threshold (Ct) values and standard deviations (± SD) of nine selected differentially expressed genes (DEGs) and the reference gene across all biological replicates in the Mock and Vln *Emilia sonchifolia* plants. Rep: Biological replicates; *GAPDH*: *glyceraldehyde-3-phosphate dehydrogenase,* used as the reference gene.

| Gene ID | Treatment | Rep 1 | Rep 2 | Rep 3 | Rep 4 | Rep 5 | Mean Ct (± SD) |
| --- | --- | --- | --- | --- | --- | --- | --- |
| *transcript_4436* | Mock | 33.0 | 33.4 | 33.0 | 32.7 | 32.2 | 32.8±0.4 |
|  | Vln | 32.6 | 32.5 | 34.7 | 34.9 | 35.8 | 34.1±1.5 |
| transcript_13717 | Mock | 29.8 | 30.5 | 28.8 | 29.7 | 29.8 | 29.7±0.6 |
|  | Vln | 29.4 | 29.1 | 31.2 | 32.1 | 33.2 | 31.0±1.8 |
| transcript_3504 | Mock | 33.5 | 33.8 | 32.8 | 33.4 | 33.7 | 33.5±0.4 |
|  | Vln | 33.7 | 33.0 | 34.7 | 35.2 | 35.9 | 34.5±1.2 |
| transcript_21885 | Mock | 26.9 | 27.9 | 28.0 | 27.5 | 27.2 | 27.5±0.5 |
|  | Vln | 22.6 | 22.5 | 24.8 | 25.5 | 26.4 | 24.4±1.8 |
| transcript_8284 | Mock | 31.3 | 32.2 | 32.7 | 32.2 | 32.1 | 32.1±0.5 |
|  | Vln | 31.1 | 31.7 | 33.0 | 33.8 | 34.0 | 32.7±1.3 |
| transcript_20375 | Mock | 27.8 | 27.5 | 26.6 | 26.2 | 26.2 | 26.8±0.7 |
|  | Vln | 24.9 | 24.8 | 26.5 | 27.4 | 28.0 | 26.8±1.2 |
| transcript_9090 | Mock | 30.9 | 31.0 | 30.9 | 30.9 | 31.1 | 30.9±0.1 |
|  | Vln | 30.1 | 30.2 | 32.9 | 33.3 | 33.1 | 31.9±1.6 |
| transcript_11781 | Mock | 28.9 | 29.7 | 29.2 | 29.5 | 29.1 | 29.3±0.3 |
|  | Vln | 25.2 | 25.4 | 27.0 | 27.9 | 27.7 | 26.7±1.3 |
| transcript_21590 | Mock | 25.4 | 25.9 | 25.9 | 25.4 | 25.8 | 25.7±0.2 |
|  | Vln | 21.3 | 21.0 | 22.2 | 22.9 | 23.7 | 22.2±1.1 |
| *GAPDH* | Mock | 30.4 | 31.2 | 30.2 | 30.0 | 30.5 | 30.5±0.5 |
|  | Vln | 30.0 | 29.9 | 32.0 | 32.8 | 33.3 | 31.6±1.6 |

**Table S6**. Mean cycle threshold (Ct) values and standard deviations (± SD) of nine selected differentially expressed genes (DEGs) and the reference gene across all biological replicates in leaf, stem, and flower tissues of Vln *Emilia sonchifolia* plants. Rep: Biological replicates; *GAPDH*: *glyceraldehyde-3-phosphate dehydrogenase*, used as the reference gene.

| Gene ID | Tissue | Rep 1 | Rep 2 | Rep 3 | Mean Ct (± SD) |
| --- | --- | --- | --- | --- | --- |
| transcript_4436 | Leaf | 34.1 | 34.3 | 35.2 | 34.5±0.6 |
|  | Stem | 33.9 | 33.0 | 33.7 | 33.6±0.5 |
|  | Flower | 31.4 | 29.5 | 29.9 | 30.2±1.0 |
| transcript_13717 | Leaf | 29.9 | 31.5 | 31.1 | 30.8±0.9 |
|  | Stem | 31.3 | 30.9 | 31.0 | 31.1±0.2 |
|  | Flower | 33.2 | 33.7 | 33.0 | 33.3±0.4 |
| transcript_3504 | Leaf | 31.8 | 33.6 | 33.5 | 32.9±1.0 |
|  | Stem | 34.4 | 33.7 | 33.4 | 33.8±0.5 |
|  | Flower | 33.8 | 32.9 | 33.3 | 33.3±0.5 |
| transcript_21885 | Leaf | 26.4 | 27.9 | 27.2 | 27.2±0.8 |
|  | Stem | 31.1 | 30.0 | 29.9 | 30.3±0.6 |
|  | Flower | 30.2 | 29.4 | 29.8 | 29.8±0.4 |
| transcript_8284 | Leaf | 32.7 | 33.7 | 33.8 | 33.4±0.6 |
|  | Stem | 31.6 | 29.7 | 31.6 | 30.9±1.1 |
|  | Flower | 30.9 | 29.8 | 29.4 | 30.0±0.8 |
| transcript_20375 | Leaf | 24.5 | 24.9 | 24.2 | 24.5±0.3 |
|  | Stem | 24.1 | 22.7 | 23.1 | 23.3±0.7 |
|  | Flower | 21.1 | 20.9 | 20.1 | 20.7±0.5 |
| transcript_9090 | Leaf | 30.1 | 32.4 | 30.2 | 30.9±1.3 |
|  | Stem | 31.0 | 30.3 | 31.1 | 30.8±0.4 |
|  | Flower | 31.6 | 29.5 | 30.7 | 30.6±1.1 |
| transcript_21590 | Leaf | 26.0 | 25.8 | 25.4 | 25.7±0.3 |
|  | Stem | 25.5 | 24.0 | 24.8 | 24.8±0.8 |
|  | Flower | 30.0 | 30.1 | 30.0 | 30.0±0.1 |
| transcript_11781 | Leaf | 35.5 | 35.2 | 35.3 | 35.4±0.2 |
|  | Stem | 35.9 | 35.5 | 35.8 | 35.7±0.2 |
|  | Flower | 36.0 | 35.1 | 35.8 | 35.6±0.5 |
| *GAPDH* | Leaf | 30.5 | 32.1 | 31.3 | 31.3±0.8 |
|  | Stem | 31.0 | 29.3 | 30.3 | 30.2±0.7 |
|  | Flower | 29.2 | 27.4 | 27.6 | 28.1±1.0 |


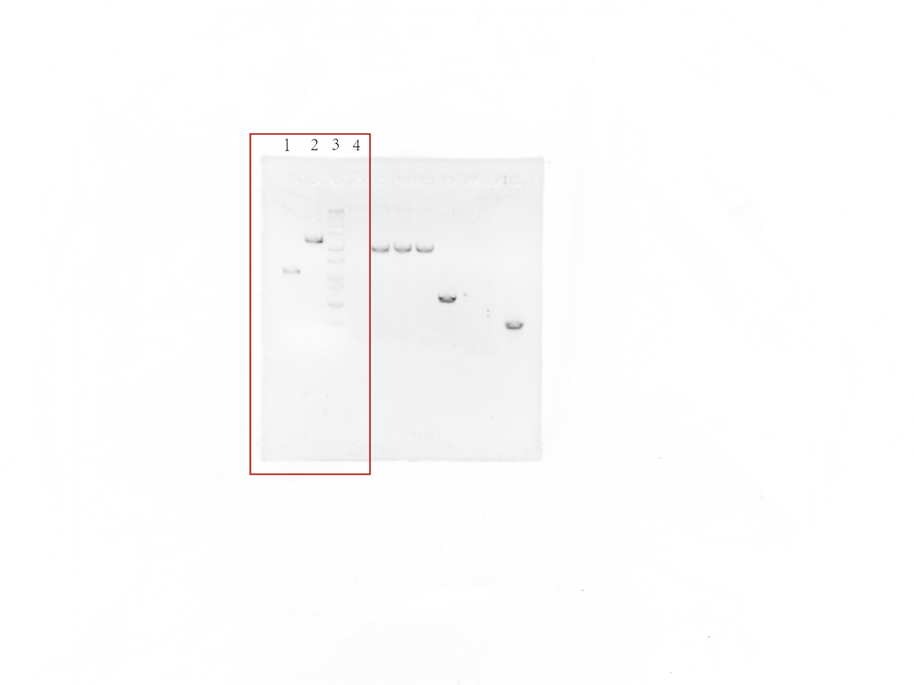


**Figure S1. Representative agarose gel electrophoresis of EYVV detection in symptomatic (Vln) and asymptomatic (Mock) plants.** Lanes 1–2 show amplification of Emilia yellow vein virus (EYVV)-associated DNA-β (~1,211 bp) and viral genomic DNA (~2,499 bp) from symptomatic (Vln) plants, as presented in Figure 1B (highlighted in red). Lane 3 contains the DNA ladder. Lane 4 represents asymptomatic (Mock) plants, in which no specific amplification bands were detected. Lane 5 and onward are unrelated reactions and are not relevant to the analysis presented.


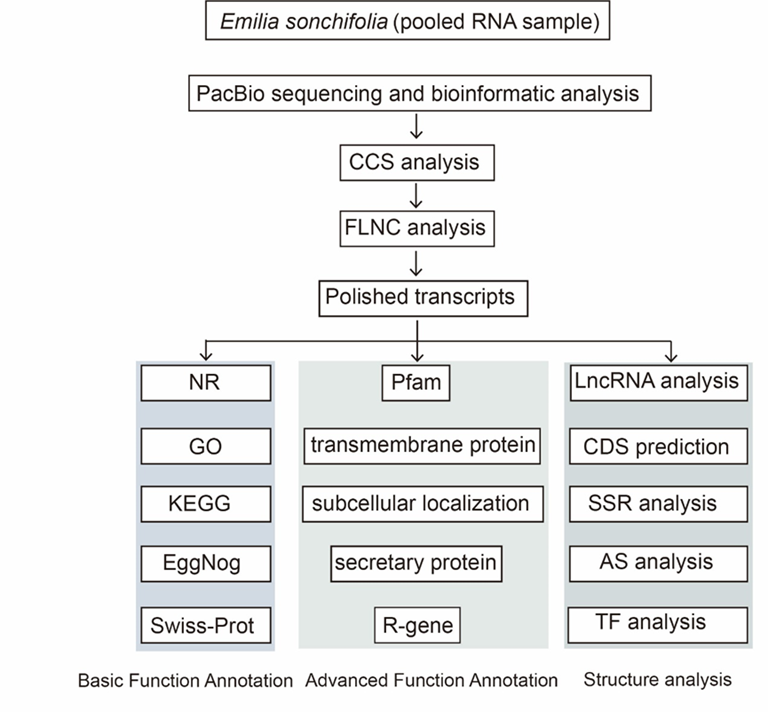


**Figure S2.** Workflow of PacBio full-length transcriptome sequencing and bioinformatic analysis in *Emilia sonchifolia*.

Pooled RNA samples were sequenced using the PacBio platform and processed through circular consensus sequence (CCS) and full-length non-chimeric (FLNC) analyses to generate polished transcripts. These transcripts were subjected to basic functional annotation (NR, GO, KEGG, EggNOG, Swiss-Prot), advanced functional annotation (Pfam, transmembrane proteins, subcellular localization, secretory proteins, and resistance genes), and structural analyses, including long non-coding RNA (lncRNA) identification, coding sequence (CDS) prediction, simple sequence repeat (SSR) analysis, alternative splicing (AS) analysis, and transcription factor (TF) identification.


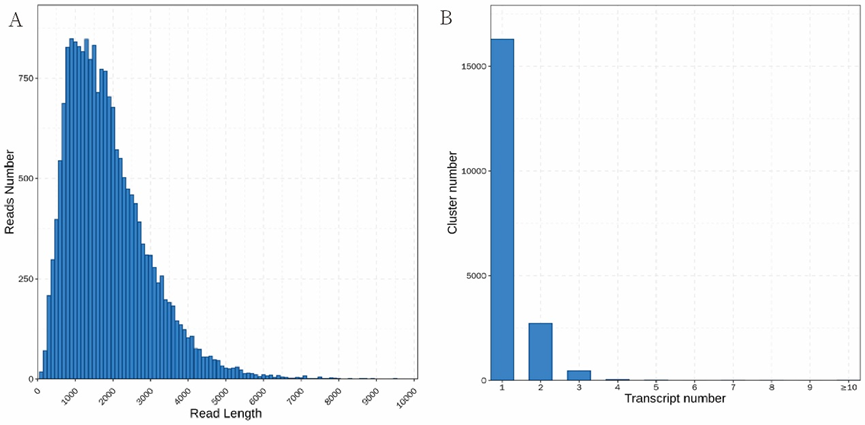


**Figure S3**. Length distribution and clustering of consensus isoforms from Single-Molecule Real-Time (SMRT) sequencing. (A) Length distribution of consensus isoforms. (B) Clustering of consensus isoforms based on the number of supporting transcripts.


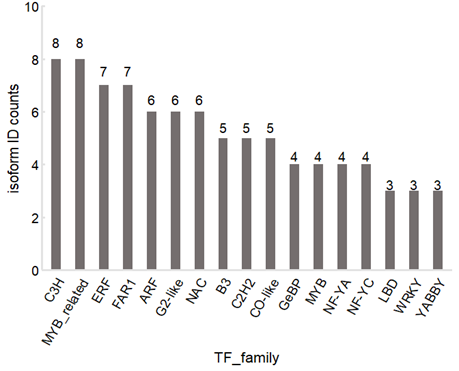


**Figure S4.** Differential expression of transcription factor (TF) families in response to virus infection in Vln plants. A total of 88 differentially expressed TF-related genes (DEGs) were identified across 17 TF families. The figure shows the number of TF members belonging to each family, illustrating the distribution of TFs in response to viral infection.


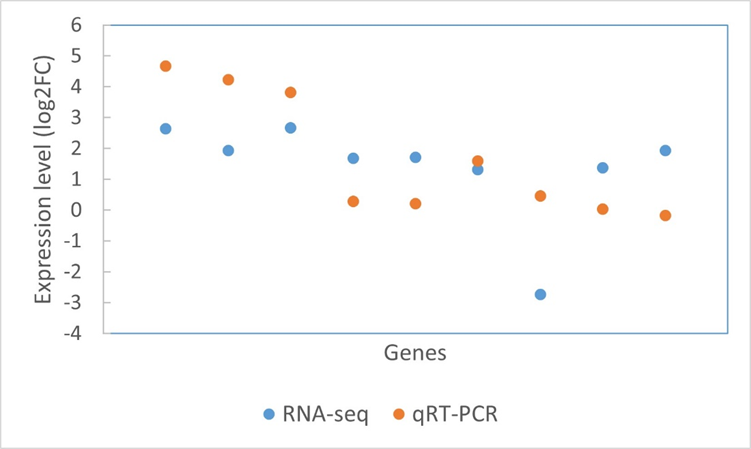


**Figure S5.** Comparison of RNA-seq and qRT-PCR log_2_ fold change values for nine selected differentially expressed gene (DEGs). Pearson correlation analysis revealed a weak positive correlation between the two datasets (r = 0.43, p = 0.252), indicating a general trend consistency between RNA-seq and qRT-PCR results.
